# Infrared radiative switching with thermally and electrically tunable transition metal oxides-based plasmonic grating

**DOI:** 10.1038/s41598-023-30959-4

**Published:** 2023-03-06

**Authors:** Ken Araki, Richard Z. Zhang

**Affiliations:** 1 Department of Mechanical Engineering, University of North Texas, Denton, 76207 USA; 2 School for Engineering of Matter, Transport & Energy, Arizona State University, Tempe, AZ 85287 USA

**Keywords:** Optics and photonics, Applied optics, Mid-infrared photonics

## Abstract

Plasmonic and phase transition has been blended to gain the infrared radiative switching which is tunable with temperature or voltage supply. This is applied via vanadium dioxide, tungsten trioxide, and molybdenum trioxide as transition metal oxides (TMO). The metallic phase at high temperature or colored state contributes in magnetic polariton (MP) excitation, producing broad absorptance. The TMO-based sub-layer is integrated underneath the grating fully supporting MP resonance. In contrast, this underlayer leads to producing the narrowband absorptance originated from concept of zero contrast grating (ZCG). The zero gradient in refractive index at the output plane of the grating cause transmission of light in broad wavelength range. With introduction of reflective silver underlayer, those transmitted through the grating are reflected back. However, there exists the near-zero narrowband transmission peaks in ZCG. This undergoes transformation to narrowband absorptance. In addition, another absorptance peak can be induced due to phonon modes at insulating phase. The MP resonance at metallic phase is characterized with inductor-capacitor (LC) circuit and the narrowband absorptance peaks are characterized with phase shift from the Fabry–Perot round trip (FP-RT) eigenequation from high contrast grating (HCG). The work expands the usage of transition metal oxides in infrared region with larger contrast.

## Introduction

The modern technology on space cooling and heating relies on electricity to modulate the temperature within residential buildings and automobiles^1^. The optical radiative emission switching is an idea to automatically tune the emission spectra depending on the environment (i.e. temperature) so that thermal management within the enclosed space can be achieved without the use of electricity^2,3^. This idea of a self-adaptive radiative cooler was proposed using phase transition material, vanadium dioxide (VO_2_) where high infrared reflectance at low temperature and high infrared emittance at high temperature is achieved^4^. VO_2_ is known to undergo phase transition at 341 K such that band structure change from monoclinic phase at low temperature to rutile phase at hot temperature, producing electrical conductivity^5^. This insulating to metallic transition has been discovered which is due to large phonon entropy production^6^. Thus, most importantly, this lattice structure change cause change in dielectric function from Lorentz oscillation to Drude model which is the explicit cause to creating optical radiative switching. This switching in refractive index is observed in the near- to far-infrared wavelength range, which exhibit near-zero extinction coefficient at insulating phase^7,8^.

By utilizing VO_2_, it can attain contrast in emittance spectrum between cold and hot state. Since VO_2_ behaves as insulator or metal depending on the temperature, infrared switching can be achieved via sandwiching VO_2_ with metallic material or sandwiching dielectric with VO_2_ to create metal–insulator-metal (MIM) structure. The methods include utilizing magnetic resonance^9–11^, Fabry–Perot dielectric cavity^12–17^, and MIM grating^18–21^ for both broadband and narrowband infrared optical switching. It relies on resonances produced within the VO_2_ cavity or dielectric cavity to capture the turn-down in optical properties. Although, some of which are designed specifically for radiative cooling do not effectively perform as a passive radiative cooler because of higher transition temperature which is higher than room temperature. To resolve the issue, one can dope tungsten to VO_2_ to lower its transition temperature but with smaller infrared switching window^22^. Alternatively, electronically tunable metal oxides, known as electrochromic materials such as tungsten trioxide (WO_3_) and molybdenum trioxide (MoO_3_) can replace VO_2_ due to its tunability with smaller voltage supply^23,24^.

Electrochromic materials are often utilized in color tuning because of their refractive index switching in visible regions. Hence, by applying the voltage (only 2 ~ 3 DC voltage), it transform from bleached to colored state with Li^+^ and H^+^ ion insertion^25,26^. Such coatings are structured in multilayers such as using Fabry–Perot WO_3_ cavity to manipulate the film color^27^. Additionally, the wavelength range can be extended to near-infrared region with different oxide, Li_4_Ti_5_O_12_ to obtain larger contrast in emittance^28^. In contrast, the optical mid-infrared switching is still limited in number of studies compared to visible region using electrochromic materials^29,30^. Recent study demonstrated the tunability in mid-infrared region using amorphous WO_3_ (aWO_3_) and crystalline WO_3_ (cWO_3_)^31^. Yet, these coatings have lower tunability compared to previously studied VO_2_ coatings.

The work proposes a new method to overcome the issue in limited usage of electrochromic metal oxides in infrared absorptance/emittance switching using plasmonic gratings. Traditionally, plasmonic gratings are introduced to excite absorptance in infrared region with optimized grating size when it is made of electron rich metal. The absorptance result from surface plasmon polariton (SPP) along the surface and magnetic plasmon polariton (MP) within the grating^32,33^. MP are induced through resonant electromagnetic coupling and conduction in deep groove cavities between metal gratings^31,32^. MP can also be induced through ceramic material, silicon carbide due to effect of surface phonon polariton (SPhP)^34,35^. Furthermore, with 2D materials such as flat graphene on plasmonic grating, the absorptance is enhanced where strong coupling between MP and SPP resonance can be observed^36,37^. Although, its absorptance can be deduced or shifted considering the effect of temperature difference during the fabrication of graphene on the grating, creating graphene wrinkles. This affects its resonance excitation and shifts its peaks but also produce surface plasmon along the wrinkled graphene^38,39^. It shows that with change in geometry of the structure, it can modulate the plasmonic resonance. However, precise control of geometry such as folding graphene is difficult.

Here, the Transition Metal Oxide (TMO) is combined with plasmonic grating so that it can produce MP resonance to induce absorptance at their metallic state where higher temperature or voltage is applied. The hybridization of MP resonance using plasmonic grating and phase transition has been previously proposed for transmittance switching, where MP assisted transmittance in achieved through unrealistic slit arrays^40^. It requires air/vacuum spacing to support MP resonance and the challenge lies in its complexity in fabrication. Compared to this work, our work is aimed to create high absorptance turn-down with TMO which is tunable thermally or electrically with simplified plasmonic grating structure. The larger absorptance is achieved through developed TMO grating compared to metal grating. In contrast, at lower temperature or at bleached state, the absorptance is not produced due to insulating phase. This replaces the conventional method to obtain MP resonance but also adds the capability to turn on and off the radiative performance such that it can adapt to the environmental condition. This work demonstrates large switching in emissive power using VO_2_, cWO_3_, and MoO_3_ by utilizing easily fabricable grating size compared to traditional deep plasmonic grating. MP resonances as a broadband absorptance are described with well-established inductor-capacitor (LC) circuit. Moreover, the new concept to obtain narrowband absorptance (broader reflectance) at insulating phase is introduced.

## Results

**Figure 1:**
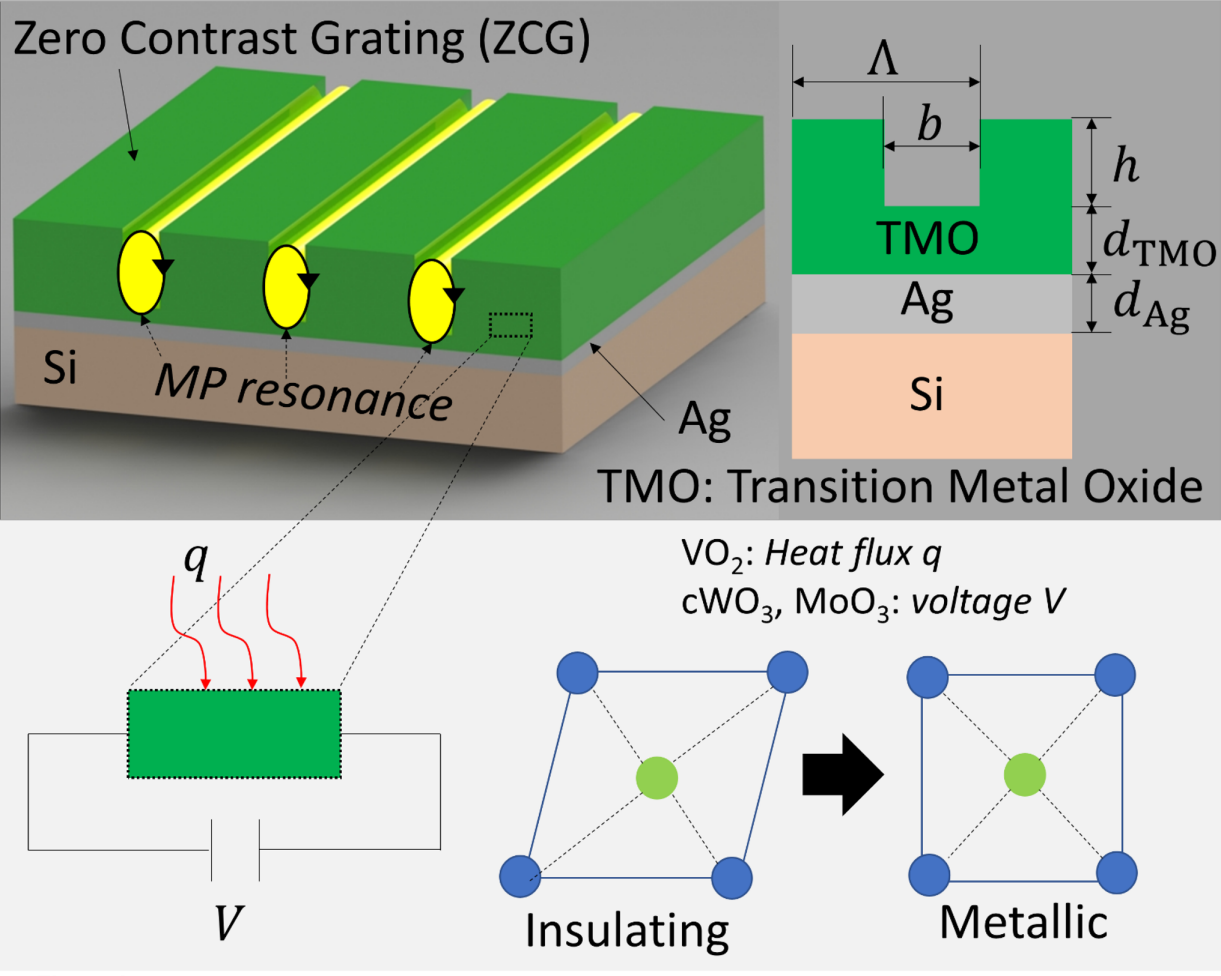
Schematic of transition metal oxide (TMO) grating with period Λ, height *h* and width *b* on TMO underlayer (*d*_TMO_) and Ag underlayer (*d*_Ag_). TMO undergoes phase transition from insulating phase to metallic phase when temperature is applied (VO_2_) or voltage is applied (cWO_3_ and MoO_3_).

Figure 1 shows the schematic of TMO grating with period Λ = 2.0 μm, height *h* = 1.0 μm, and *b* = 0.5 μm on TMO underlayer with *d*_TMO_ = 0.2 μm and Ag underlayer with *d*_Ag_ = 0.2 μm. At low temperature or without any voltage supply, the TMO underlayer behaves as zero contrast grating (ZCG) which transmits light in broader wavelength ranges. The transmitted light is reflected at the interface of 0.2 μm thick Ag underlayer so that broader reflectance can be obtained. Ag layer is also important to ensure the opaqueness at insulating phase. ZCG is a geometric method which was introduced to capture broad reflectance compared to high contrast grating (HCG)^41–44^. As temperature or voltage is applied, the monolithic lattice structure transitions to atomically ordered rutile phase. Due to the metallic feature of TMO at hot state or colored state, the selected geometric size can excite magnetic polariton (MP) resonance to induce sharp absorptance peak. 0.2 μm-thick TMO underlayer is introduced under the TMO strip which is enough for dissipation thickness of about *δ* = *λ*/4π*κ* = 0.11, 0.19 and 0.1 μm (for VO_2_, cWO_3_, and MoO_3_ respectively at 1550 cm^−1^) to fully support MP resonance.

**Figure 2:**
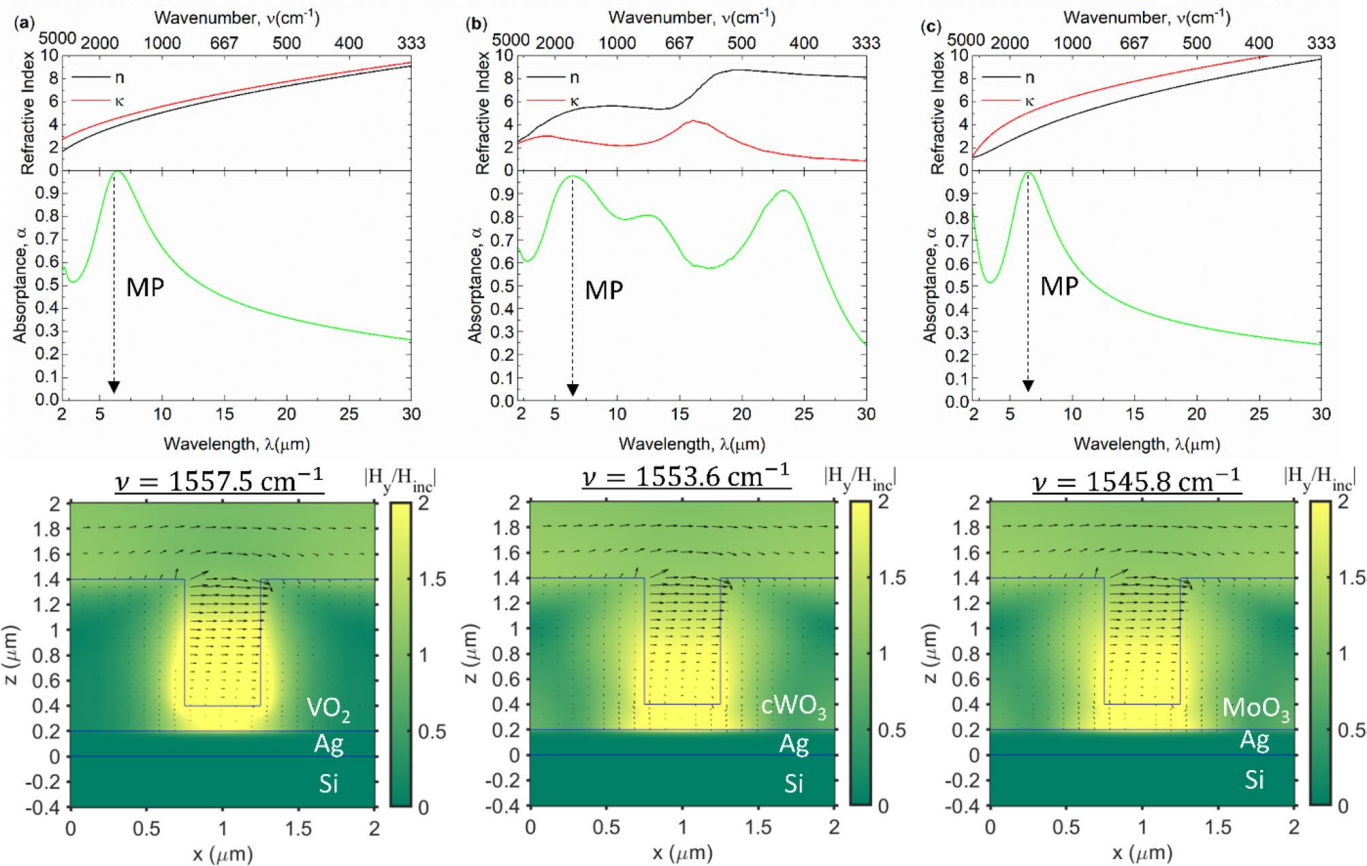
Refractive index *n*, *κ*, absorptance spectrum, and electromagnetic field contour of (**a**) VO_2_, (**b**) cWO_3_, and (**c**) MoO_3_ at metallic (colored) state. The magnetic field is shown in contour and black array shows the electric field vectors.

For the metallic phase of TMO, the first order MP resonance can be obtained within the 1 μm thick groove. Consequently, near-unity normal incident absorptance at TM wave is achieved as shown in Fig. 2 for (a) VO_2_, (b) cWO_3_, and (d) MoO_3_ respectively. As can be indicated from the absorptance spectrum, the MP1 resonance frequency is centered around 1555 cm^−1^ because of its similar plasma frequency of $2.57\times {10}^{15}$ to $2.7\times {10}^{15}$ rad/s^45,46^. Contrary, silver does not excite MP resonance with selected grating size because it has plasma frequency of $1.39\times {10}^{16}$ rad/s^33^. Note that the dielectric function of VO_2_ and MoO_3_ at metallic phase is designed with Drude model. In contrast, cWO_3_ is taken from the experimental study which shows the Drude-Lorentz characteristic in mid-infrared as shown in the refractive index curve. Thus, cWO_3_ has an effect of Lorentz oscillation at colored state resulting in broader absorptance. As a result, higher emissive power of 622 W/m^2^ can be gained when voltage is applied to cWO_3_ based grating. On the other hand, VO_2_ and MoO_3_ have pure MP resonance excitation which results in emissive power of 488 and 454 W/m^2^ (at 350 K) respectively. Therefore, the electromagnetic field distribution shows electromagnetic loop within the grating which is the major characteristic of MP resonance.

**Figure 3:**
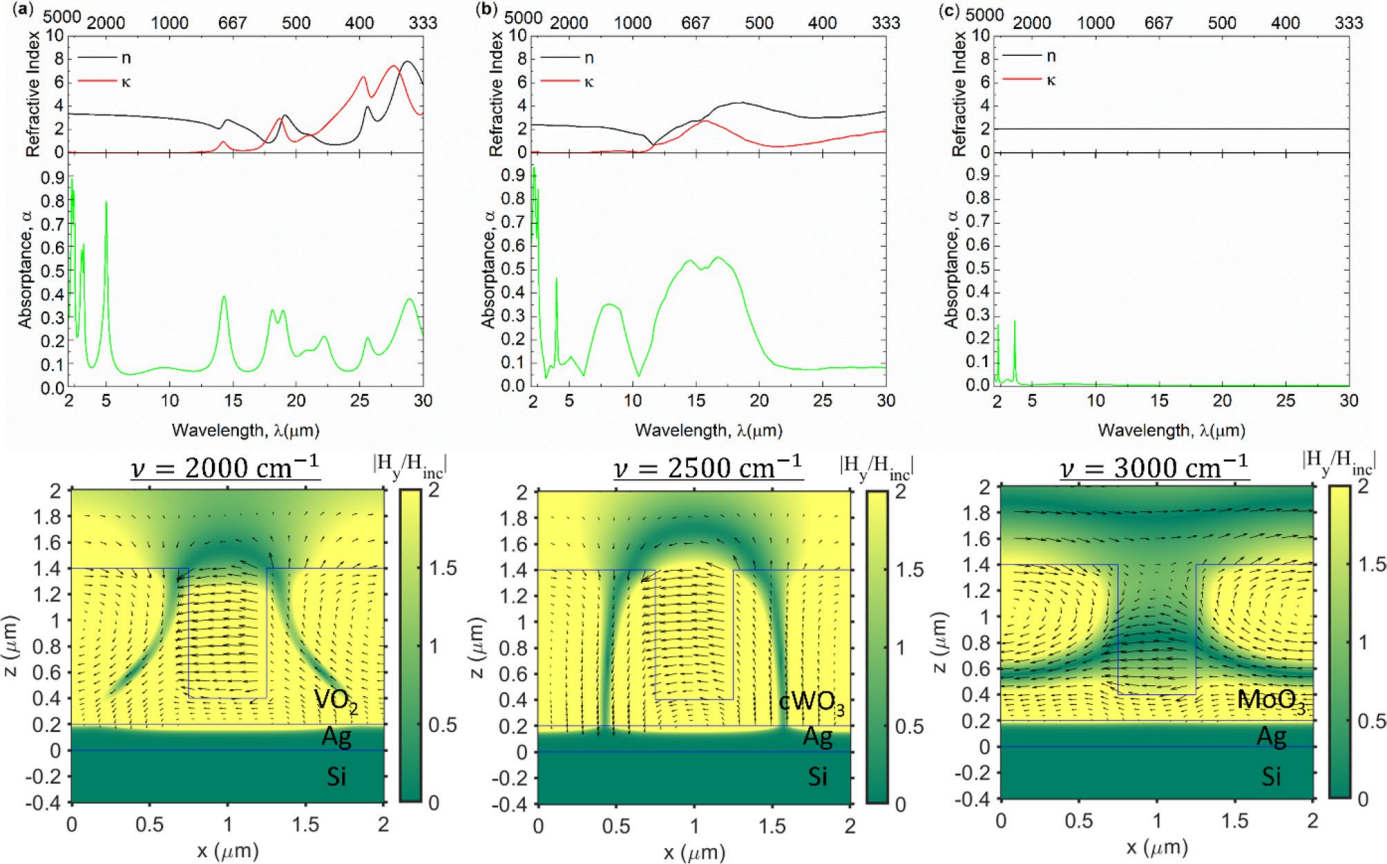
Refractive index *n*, *κ*, absorptance spectrum, and electromagnetic field contour of (**a**) VO_2_, (**b**) cWO_3_, and (**c**) MoO_3_ at insulating phase (bleached) state. The magnetic field is shown in contour and black array shows the electric field vectors.

At the low temperature and bleached state, TMO becomes transparent in infrared region with near zero extinction coefficient as shown in refractive index plot in Fig. 3. This is a key feature in TMO which directly results in obtaining the optical switching in infrared region. As can be indicated from the absorption spectrum, VO_2_ and cWO_3_ have multiple absorption peaks because of Lorentz oscillation existence beyond 10 μm while MoO_3_ have small absorption peaks due to transparency in wider range of infrared light. Thus, with and without phonon contribution, the difference in absorption at insulating phase can be observed. First, when the refractive index of the grating strips and the underlayer is the same, but also with near zero extinction coefficient, the light transmits through the grating without disturbance. This is known as a characteristic of zero contrast grating (ZCG) which is an opposite structure to the high contrast grating (HCG). HCG is a grating made of high-index material surrounded by low index medium to obtain high reflectance^43,47^. By applying ZCG on the reflective material, the transmitted light is reflected back to create a perfect mirror in wider range of wavelength compared to HCG. Hence, the reflection band can be directly converted to zero absorptance because of opaqueness (*α* = 1 – *ρ*), whereas the narrowband transmittance due to waveguide interference (even waveguide mode) is converted as a narrowband absorptance. This is the case for non-Lorentz material such as MoO_3_. Second, with Lorentz oscillation, the reflection band is split into two where two different waveguide mode (single and even waveguide mode) is excited, creating a dual-mode region, similar to HCG. Consequently, multiple absorption peaks are produced because of these two waveguide modes. Therefore, cWO_3_ and VO_2_ have combined effect of ZCG and phonon modes, resulting in mix of two transmission modes split with the presence of phonon. As shown in the electromagnetic field distribution, it results in confining electromagnetic fields both in grooves and gratings. On the contrary, MoO_3_ only has electromagnetic field confinement in the grating as an even waveguide mode, which result in small absorption peaks. This tells how transparency throughout a wide range of infrared light is significant to obtain broader reflectance using ZCG. The emissive power at insulating phase is 51.6 and 116 W/m^2^ (at 300 K) for VO_2_ and cWO_3_ while MoO_3_ only has 3.15 W/m^2^. Therefore, as summarized in Table 1, MoO_3_ can achieve emissive power ratio of 144 with absorptance difference Δα of 0.972 at MP1 frequency. This is much larger than other TMO.

**Table 1:** Absorptance difference and emissive power ratio of TMO grating.

TMO	MP frequency	α_MP_	Insulating (bleached)	Δα	*P*_m_ (W/m^2^)	*P*_i_ (W/m^2^)	*P*_m_/*P*_i_
VO_2_	1557.5 cm^−1^	0.9436	0.0564	0.8872	488	51.6	9.46
cWO_3_	1553.6 cm^−1^	0.8627	0.1151	0.7476	622	116	5.38
MoO_3_	1545.8 cm^−1^	0.9832	0.0112	0.972	454	3.15	144

## Discussions

**Figure 4:**
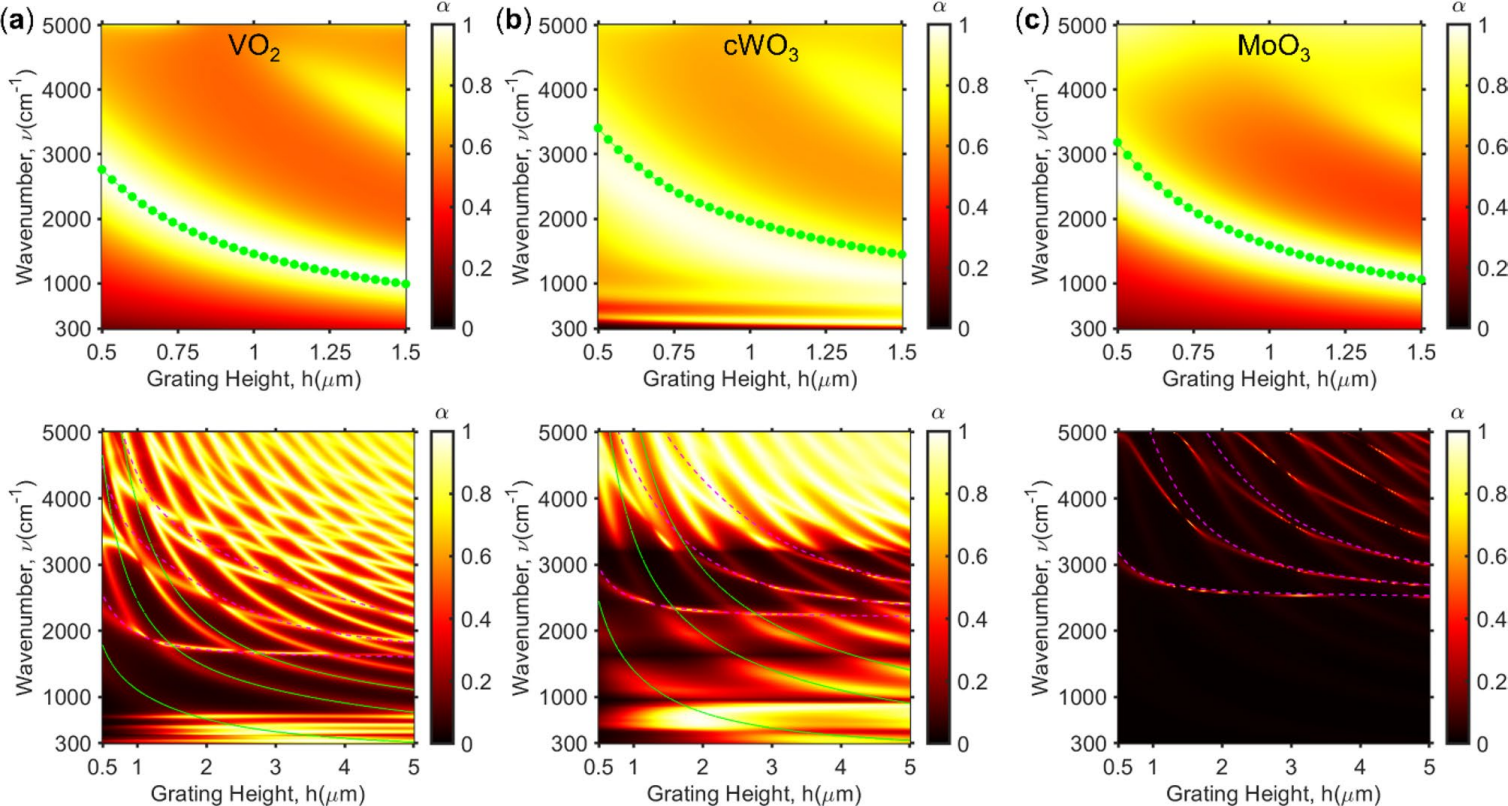
Absorptance contour in respect with grating height and wavenumber at metallic state (upper row) and insulating phase (lower row) for (**a**) VO_2_, (**b**) cWO_3_, and (**c**) MoO_3_.

Figure 4a–c represents the absorptance contour in respect with the grating height at metallic phase and insulating phase for VO_2_, cWO_3_, and MoO_3_. The MP resonance frequency can be described with inductor (*L*) – capacitor (*C*) circuit where TMO trench walls behave as inductor and gap behave as capacitor. The resonance frequency is determined by $1/\surd LC$. The inductor is given by,

1 $$L = \mu_{0} hb - \frac{2h + b}{{\varepsilon_{0} \omega^{2} \delta }}\frac{{\varepsilon^{{\prime}{2}} }}{{\varepsilon^{{\prime}{2}} + \varepsilon^{{\prime\prime}{2}} }},$$

where *ε*’ and *ε*’’ are the real and imaginary part of the permittivity. *Ε*_0_ and *μ*_0_ are the vacuum permittivity and permeability respectively^48^. The inductor is highly dependent on the dielectric function, and it is inversely proportional to the MP frequency. Thus, the difference in plasma frequency is significant. The capacitance is expressed by,

2 $$C = c^{\prime}_{{{\text{gap}}}} \frac{{\varepsilon_{0} h}}{b},$$

where *c*’_gap_ = 1.0 is the numerical factor^48^. By utilizing the LC circuit model, the MP resonance frequency is predicted as shown in green solid lines in upper row of Fig. 4. The curve matches well in the region with near unity absorptance for each material. The predicted curve follows a bit off the maximum absorption for cWO_3_ because of the Drude-Lorentz type dielectric function. With increase in grating height, the MP peaks shifts toward far infrared region due to inverse proportionality. It can also produce multiple MP modes for deeper grooves.

As shown in the lower row of Fig. 4, the narrowband absorptance can be observed because of ZCG but also the presence of phonons for VO_2_ and cWO_3_. Due to phonon contribution, the single waveguide mode is strongly present. This can be described with Fabry–Perot round trip (FP-RT) eigenequation from HCG as,

3 $${\mathbf{M}}\left( {\lambda ,h} \right)\left[ {\begin{array}{*{20}c} {A_{0} } \\ {A_{2} } \\ \end{array} } \right] = \left| Q \right|e^{i\phi } \left[ {\begin{array}{*{20}c} {A_{0} } \\ {A_{2} } \\ \end{array} } \right],$$

where **M** is the RT propagation matrix, *A*_j_ is the eigenmode expansion coefficients, |*Q*|*e*^i*ϕ*^ is the eigenvalues, and *ϕ* is the phase condition^43,47^. The dispersion relation from the matrix equation for normal incidence can be determined by,

4 $$k_{{{\text{ZCG}},m}} \tan \left( {\frac{{k_{{{\text{ZCG}},m}} f\Lambda }}{2}} \right) + n_{{{\text{ZCG}}}}^{2} k_{{{\text{air}},m}} \tan \left[ {\frac{{k_{{{\text{air}},m}} \left( {1 - f} \right)\Lambda }}{2}} \right] = 0,$$

where *k*_ZCG_ and *k*_air_ represent wavevector in *x*-direction within the grating and air (trench), respectively^44,47^. These are based on HCG where grating is surrounded by low-index material. The same can be applied to ZCG, itself. However, with ZCG on silver underlayer, the phase change must be considered between the reflection ray between the standing ZCG and ZCG on silver layer. This is implemented to obtain the narrowband absorption observed for the insulating phase. First, the single waveguide mode with phase shift can be calculated with,

5 $$h = \frac{{\left( {2m - 1} \right)\pi }}{{2\beta_{0} \left( \omega \right)}},$$

where *β*_0_ is the longitudinal wavevector for single waveguide mode and *m* = 1, 2, 3, …. The grating height for narrowband absorption is the phase shift of -π/2*β*_0_(ω) from either HCG reflection or ZCG transmission mode. However, this does not exist without the help of phonons. Thus, only VO_2_ and MoO_3_ produce additional absorption peaks as shown in green solid lines in Fig. 4. Whereas the even waveguide mode can exist for any TMO where absorptance peak is directly converted from the near-zero transmission peaks. The even waveguide mode is also determined with a phase change of -π/2*β*_2_(ω)-π/2*β*_0_(ω) such that the grating height is calculated as,

6 $$h = \frac{\pi }{2}\left[ {\frac{2m - 1}{{\beta_{2} \left( \omega \right)}} - \frac{1}{{\beta_{0} \left( \omega \right)}}} \right]$$

Thus, the narrowband absorption observed in MoO_3_ is the even waveguide mode described by the above equation which is shown in pink dashed line. This shows how ZCG can be utilized to obtain high *Q*-factor absorptance peak at desired wavelength by adjusting the grating height *h* and period Λ when ZCG is made of transparent dielectric material. Although, the dispersion curve is also dependent on the incident angle.

**Figure 5:**
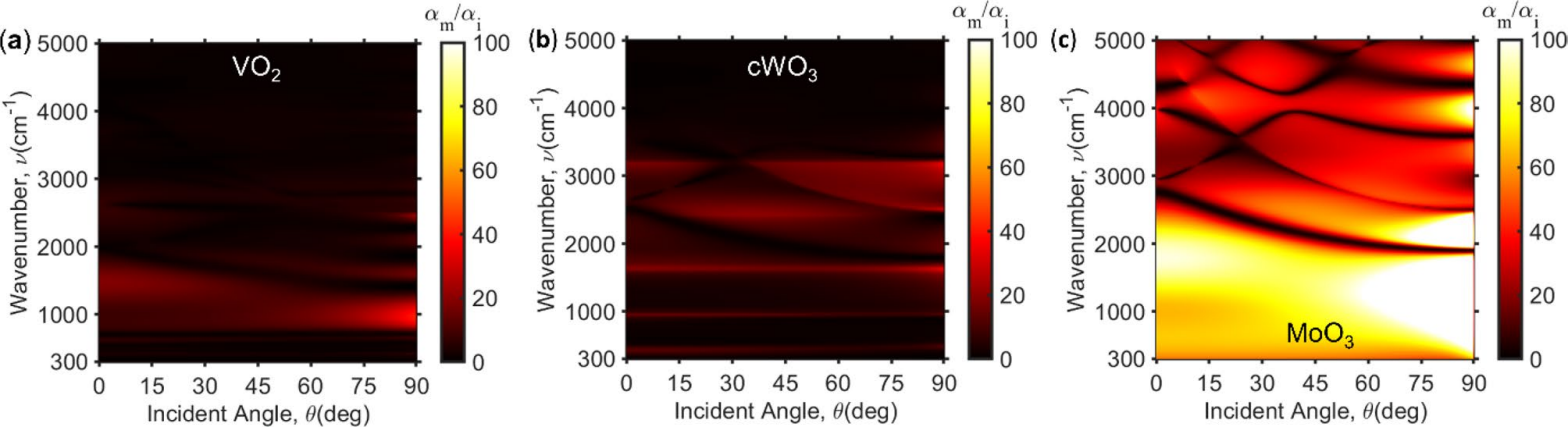
Absorptance ratio (α_m_/α_i_) contour in respect with incident angle and wavenumber for (**a**) VO_2_, (**b**) cWO_3_, and (**c**) MoO_3_.

Because of dependency in the incident angle at the insulating phase, one can obtain large contrast at certain incident angle. Figure 5a–c shows the absorptance ratio α_m_/α_i_ contour of VO_2_, cWO_3_, and MoO_3_. Due to the fact that VO_2_ and cWO_3_ have phonon modes, it results in lower contrast while MoO_3_ has the largest contrast especially at the oblique incident angle (See Fig. S1 for absorptance dependency on incident angle). This is because of the photonic band structure at the oblique angles which can be described with the Bloch condition: *k*_x,*j*_ = 2πsin*θ*/λ + 2π*j*/Λ where *j* is the diffraction orders of the periodic grating^49^. The effect of oblique incidence to the ZCG can be related to the band structure and additional odd waveguide mode should be considered which produce tri-mode region^43^. Thus, as shown in Fig. 5c, MoO_3_ in particular, have multiple narrowband regions with low absorptance ratio which relates to band structure of ZCG. In contrast, high ratio is obtained in lower wavenumber range because no existence of waveguide mode in oblique incident in addition to the second reflective band, resulting in broad reflectance from 300 to 3000 cm^−1^ at bleached state. Moreover, the transparency of MoO_3_ beyond 3000 cm^−1^ still can obtain larger ratio compared to others.

**Figure 6:**
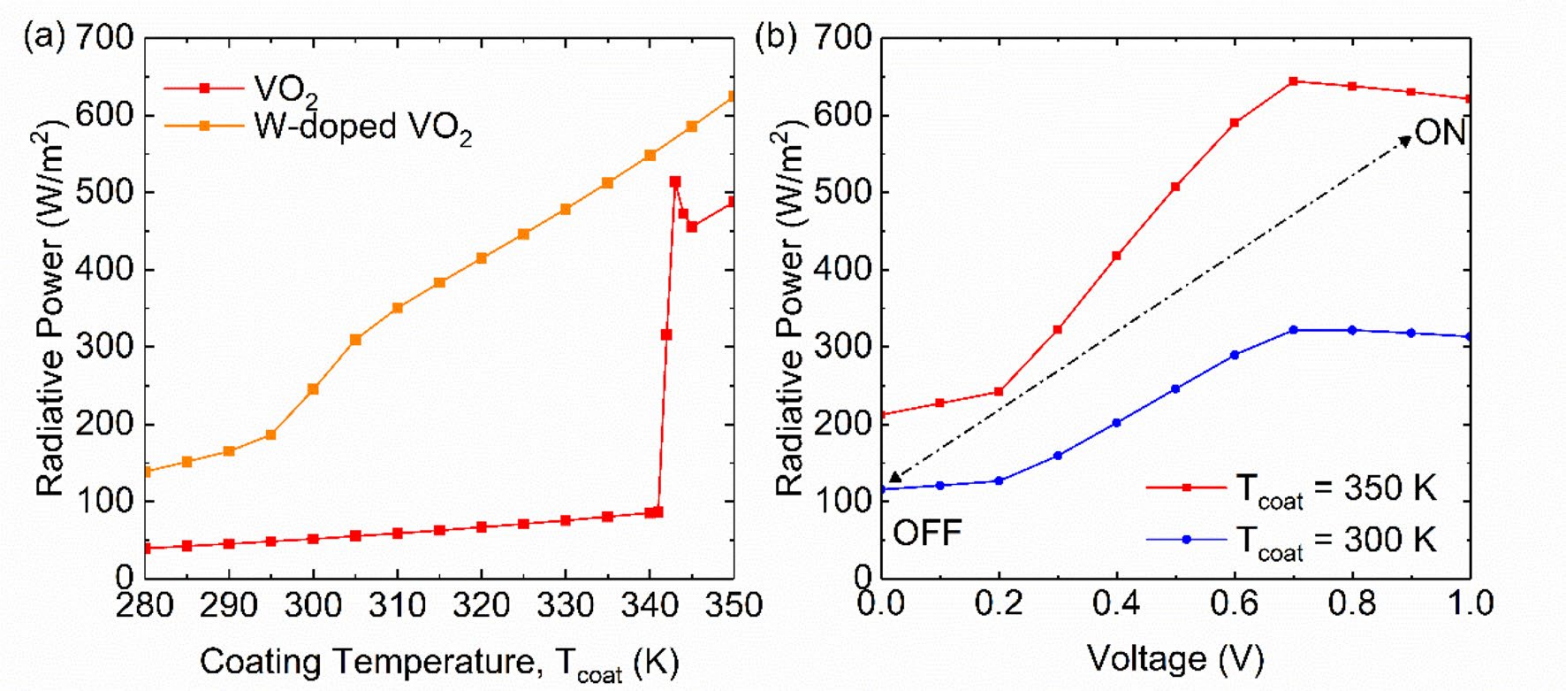
(**a**) Relationship between radiative power and coating temperature for VO_2_ and W-doped VO_2_. (**b**) Relationship between radiative power and applied voltage for cWO_3_.

Lastly, we demonstrate the performance over temperature and voltage for VO_2_ and cWO_3_ by using the Bruggeman effective medium theory expressed as,

7 $$f\frac{{\varepsilon_{{\text{m}}} - \varepsilon_{{{\text{eff}}}} }}{{\varepsilon_{{{\text{eff}}}} + q\left( {\varepsilon_{{\text{m}}} - \varepsilon_{{{\text{eff}}}} } \right)}} + \left( {1 - f} \right)\frac{{\varepsilon_{{\text{i}}} - \varepsilon_{{{\text{eff}}}} }}{{\varepsilon_{{{\text{eff}}}} + q\left( {\varepsilon_{{\text{i}}} - \varepsilon_{{{\text{eff}}}} } \right)}} = 0$$

where *q* is the depolarization factor^49^. The effective permittivity is, therefore, determined by solving zero on right hand side of the equation. Here, we also demonstrate the effect of doping tungsten to VO_2_ which shifts the transition temperature to room temperature. The filling ratio *f* and *q* for VO_2_, W-doped VO_2_ (1.63at.%) and cWO_3_ is taken and derived from the literatures (See Table S1)^22,50,51^. Figure 6 represents the result of the radiative power which is the integrated spectral emissive power *P*_λ_ = *α*_λ_*E*_BB_,_*T*_, where *E*_BB_,_*T*_ is the blackbody radiation at temperature *T*, in respect with temperature or voltage. With rise in temperature or voltage, the dielectric function undergoes transition from semiconducting Lorentz oscillating model to metallic Drude model with increase in metallic phase filling ratio *f*^16^. As a consequence, VO_2_ experience rapid increase in radiative power because of shorter transition window of about 5 K^50^. However, with tungsten doping, it results in slower increase in power because of wider transition window of about 50 K^22^. Because of larger concentration tungsten, higher absorption is expected even at insulating phase at 293 K. Therefore, overall emissive power from insulating to metallic phase is high compared to pure VO_2_. It has been examined that with lower W concentration, the extinction coefficient can be reduced at insulating phase but with increase in transition temperature^22^. In contrast, cWO_3_ have its ability to switch on and off the radiative power by applying voltage while changing its color. Depending on the environmental temperature, it can turn off the power to 100 W/m^2^ or turn on to 600 W/m^2^. Notice that the radiative power for cWO_3_ at insulating phase is relatively large since phonon modes present beyond 10 μm is the contributes in 66 to 74% in total power for 300 and 350 K respectively. Without the phonon modes, the power at low temperature or off state is expected to drop more than two-fold.

In conclusion, the work demonstrate how Transition Metal Oxide (TMO) can be utilized as a plasmonic grating by applying the temperature or voltage. The magnetic polariton (MP) resonance excitation is achieved by the metallic state of VO_2_, cWO_3_, and MoO_3_ which exhibit near unity absorption peak at around 1555 cm^−1^_._ The inductor-capacitor (LC) circuit is utilized to describe its shifting toward low wavenumber as grating height increases. While at the insulating or bleached state of TMO, two types of narrowband absorption appear as waveguide modes which relates to Fabry–Perot round trip (FP-RT) eigenequation. The transmission modes in zero contrast grating (ZCG) is converted to broad reflectance due to disturbance of FP-RT interference at the output plane of the grating. However, the near-zero transmission peaks are transformed to the narrowband absorption peaks with phase shift. For VO_2_ and cWO_3_, the existence of Lorentz oscillation in mid-infrared region assists in producing the single waveguide mode in addition to even waveguide mode. Thus, multiple absorptance peaks are generated. On the other hand, MoO_3_ only excite even mode due to absence of phonon absorption. Therefore, MoO_3_ can enhance the emissive power ratio to 144 which is much larger than other TMOs. The TMO plasmonic grating can be used for radiative switching coating for electronics, batteries, and rooftops to manipulate the radiative power with temperature and voltage as demonstrated in the relationship between the radiative power.

## Methods

The optical radiative properties of TMO grating structure are calculated using Rigorous-Coupled Wave Analysis (RCWA) which is a well-established method to solve for Maxwell Equation. Utilizing the spectral reflectance *ρ*_λ_ obtained from RCWA, the spectral absorptance is calculated as α_λ_ = 1 − *ρ*_λ_. The dielectric function of metallic phase is determined by the Drude mode as,

8 $$\varepsilon_{{\text{m}}} = \varepsilon_{\infty } - \frac{{\omega_{{\text{p}}}^{{2}} }}{{\omega \left( {\omega + i\gamma } \right)}}$$

For MoO_3_, the plasma frequency is ${\varepsilon }_{\infty }$= 5.7, *ω*_p_ = 13,642 cm^−1^, and *γ* = 2621 cm^−1^^46,52^. Similarly, the dielectric function of insulating phase is determined by Lorentz oscillation model as,

9 $$\varepsilon_{{\text{i}}} = \varepsilon_{\infty } + \sum\limits_{j = 1}^{N} {\frac{{S_{j} \omega_{j}^{2} }}{{\omega_{j}^{2} - i\gamma_{j} \omega - \omega^{2} }}}$$

The dielectric function of VO_2_ and cWO_3_ for insulating and metallic phase is taken from the literatures^7,23^. The radiative power is obtained from integrating the absorbed blackbody radiation (*E*_BB,*T*_) at certain temperature, *T* as,

10 $$P = \int_{{2{\mu m}}}^{{30{\mu m}}} {\alpha_{\lambda } E_{{{\text{BB}},T}} d\lambda }$$

In this study, the radiative power is only calculated on normal incidence since MP resonance, which only excites in TM wave, is independent of the incident angle (See upper row in Supplementary Fig. S1). The broad absorptance band is maintained up to 75 degrees of incidence, where the radiative power can be maintained above 450 W/m^2^ for VO_2_ and 600 W/m^2^ for WO_3_. Whereas the transmission mode in insulating phase is dependent on the incident angle which also applies to the transformed narrowband absorption peaks. Nonetheless, the major contributor of radiative power in insulating phase for VO_2_ and WO_3_ are the phonon modes which are independent on the incident angle, which keeps power in range of 50 to 100 W/m^2^ (See lower row in Fig. S1).

## Supplementary Information

Supplementary Information.

## Data Availability

All data generated or analyzed during this study are included in this published article and its supplementary information files.
