# Supplementary material for: Infrared radiative switching with thermally and electrically tunable transition metal oxides-based plasmonic grating
Source: Sci Rep. 2023 Mar 6;13:3702. doi: 10.1038/s41598-023-30959-4 (PMC9988874; doi:10.1038/s41598-023-30959-4)
Supplement: Supplementary file 1 — Supplementary Information. [file 41598_2023_30959_MOESM1_ESM.pdf]

Supplementary Material for

Infrared Radiative Switching With Thermally and Electrically Tunable Transition Metal Oxides-Based Plasmonic Grating

Ken Araki[1, 2*], and Richard Z. Zhang[1]

[1]Department of Mechanical Engineering, University of North Texas, Denton, 76207, USA.
[2]School for Engineering of Matter, Transport & Energy, Arizona State University, Tempe, AZ, 85287, USA
[*]karaki1@asu.edu

Absorptance spectra dependency on incident angle and polarization

Figure S1 shows the absorptance (α) contour of VO_2, cWO_3, and MoO_3 grating structures for transverse magnetic (TM) wave where upper row represents the metallic state and lower row represents the insulating phase. As can be indicated from the contour plot at metallic state, magnetic polariton (MP) resonance peak is independent of the incident angle. This is one of the characteristics of MP resonance to the incidence angle that excite only in TM wave unless there are no other resonances (i.e., SPP, SPhP) interacting with it.[1-3] Note that MP resonance excitation in TM wave is only true for 1D periodic plasmonic gratings.

On the other hand, the absorption peaks converted from near-zero transmittance peaks in zero contrast grating (ZCG) at insulating phase is highly dependent on incidence angle due to the Bloch-Floquet condition where periodicity of the grating (Λ: period) plays apart in reflection of angle of each diffraction orders, j. The reflection angle θ_j can be determined from $\sin\theta_j = \sin\theta + j\lambda/\Lambda$, where θ is the incident angle and λ is the wavelength, which also describes the incident angle dependence in high contrast grating (HCG).[4,5] This effect is also similar to SPP excitation along the periodic metallic gratings.[6] Hence, the narrowband absorptance peaks observed in ZCG on metallic film are directional, whereas MP resonance peaks are not.

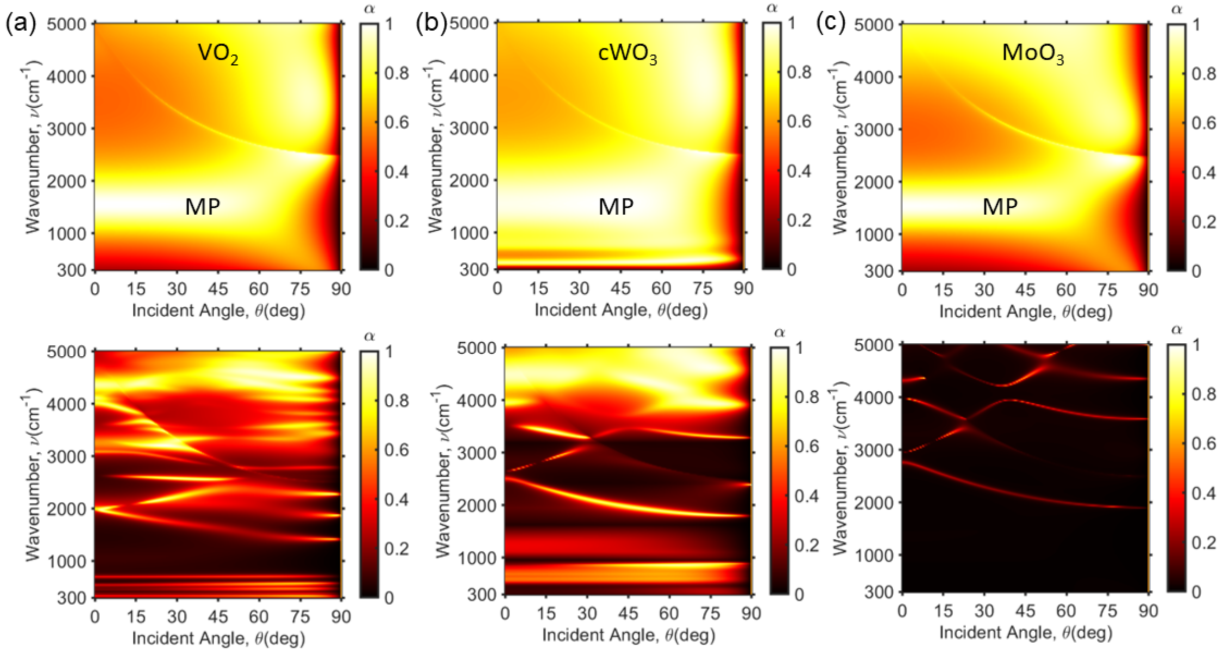

Fig. S1. Absorptance contour of (a) VO_2, (b) cWO_3, and (c) MoO_3 at metallic state (upper row) and insulating state (lower row) in TM wave.

Figure S2 represents the absorptance contour in transverse electric (TE) wave in both metallic and insulating phase of VO_2, cWO_3, and MoO_3. Since this work is 1D periodic grating, MP resonance does not excite in TE wave as shown in the upper row of the figure at the metallic phase of transition metal oxides. Thus, TE wave is not considered in this study and only focused on TM wave. Although, the narrowband absorption at insulating phase is present because the transmission mode in ZCG excites both in TM and TE wave with some difference. Due to electric field (E_y) being the assertive element in TE wave, whereas magnetic field (H_y) in TM wave, the dual-mode region is different between the polarization. Therefore, absorptance peak locations are not the same in TM and TE waves.

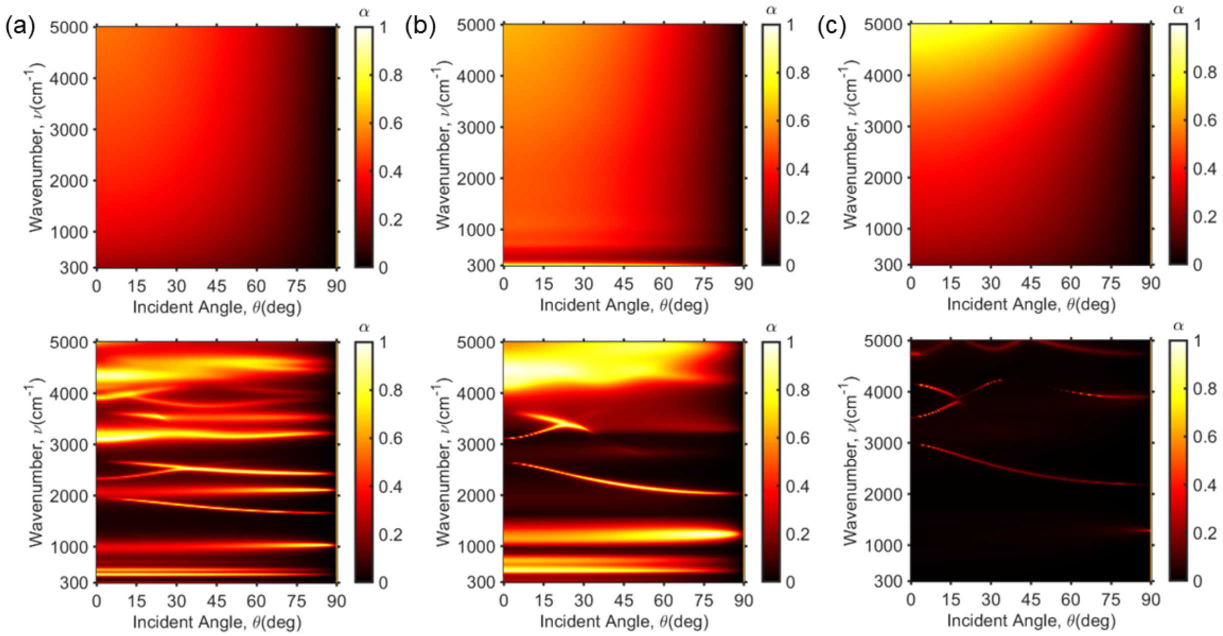

Fig. S2. Absorptance contour of (a) VO_2, (b) cWO_3, and (c) MoO_3 at metallic state (upper row) and insulating state (lower row) in TE wave.

Metal-to-insulator transition (MIT) curve estimated from Bruggeman effective medium theory (EMT)

Bruggeman EMT is utilized to obtain the MIT transition curve in terms of metallic filling ratio *f*. The Bruggeman EMT for MIT transition is expressed as,

$$f\frac{\varepsilon_{\mathrm{m}}-\varepsilon_{\mathrm{eff}}}{\varepsilon_{\mathrm{eff}}+q\left(\varepsilon_{\mathrm{m}}-\varepsilon_{\mathrm{eff}}\right)}+\left(1-f\right)\frac{\varepsilon_{\mathrm{i}}-\varepsilon_{\mathrm{eff}}}{\varepsilon_{\mathrm{eff}}+q\left(\varepsilon_{\mathrm{i}}-\varepsilon_{\mathrm{eff}}\right)}=0 \quad (1)$$

Where ε_m, ε_i and ε_{eff} are the dielectric function of metallic, insulating and effective medium. q is the depolarization factor where the values for VO_2 is taken from Ref 7 where it is considered fully insulating at 341 K and fully metallic at 345 K and values needed for Bruggeman EMT are listed in Table S1.[7] Note that the dielectric function of VO_2 is anisotropic which is taken from Ref.[8]

For cWO_3, the depolarization factor is taken as q = 0.5 due to no dependency in polarization. The filling ratio *f* is estimated from the experimental literature Ref. 8 where experimental study on relationship between voltage and refractive index is studied.[9] With known refractive index with the voltage from bleached to colored state, the filling ratio is assumed to be f = 0.0 at bleached state and f = 1.0 at colored state. Utilizing the experimentally measured refractive index, the filling ratio is estimated from Bruggeman EMT, Eq. (1). Table S2 summarizes the estimated filling ratio in relation to the voltage. Thus, the effective permittivity between metallic and insulating phase is calculated using those filling ratio and depolarization factors listed in Tables.

Table S1. The relationship between the depolarization factor q for ordinary (O) and extraordinary (E) direction and filling ratio (f) taken from Ref. 7 for VO_2.

T(K)	q_O	$q_E = 1 - 2q_O$	f
342	0.2	0.6	0.18
342.6	0.33	0.34	0.31
343	0.45	0.1	0.48
343.6	0.5	0	0.7

Table S2. The estimated filling ratio for cWO_3 from the relationship between voltage and refractive index taken from Ref. 9.

Voltage (V)	0.0	0.2	0.4	0.6	0.8	1.0
Refractive Index	2.23	2.17	1.87	1.61	1.28	1.18
f	0.0	0.0441	0.2796	0.5116	0.8694	1.0

References

1 Lee, B. J., Wang, L. P. & Zhang, Z. M. Coherent thermal emission by excirtation of magnetic polaritons between periodic strips and a metallic film. *Opt Express* **16**, 11328-11336 (2008).

2 Wang, L. P. & Zhang, Z. M. Resonance transmission or absorption in deep gratings explained by magnetic polaritons. *Appl. Phys. Lett.* **95**, doi:10.1063/1.3226661 (2009).

3 Zhao, B. & Zhang, Z. M. Study of magnetic polaritons in deep gratings for thermal emission control. *J Quant Spectrosc Radiat Transf* **135**, 81-89, doi:10.1016/j.jqsrt.2013.11.016 (2014).

4 Qiao, P., Yang, W. & Chang-Hasnain, C. J. Recent advances in high-contrast metastructures, metasurfaces, and photonic crystals. *Advances in Optics and Photonics* **10**, doi:10.1364/aop.10.000180 (2018).

5 Zhang, Z. M. *Nano/microscale heat transfer*. (Springer, 2020).

6 Chen, Y. B. & Zhang, Z. M. Design of tungsten complex gratings for thermophotovoltaic radiators. *Opt. Commun.* **269**, 411-417, doi:10.1016/j.optcom.2006.08.040 (2007).

7 Qazilbash, M. M. *et al.* Infrared spectroscopy and nano-imaging of the insulator-to-metal transition in vanadium dioxide. *Phys. Rev. B* **79**, doi:10.1103/PhysRevB.79.075107 (2009).

8 Barker, A. S., Verleur, H. W. & Guggenheim, H. J. Infrared Optical Properties of Vanadium Dioxide Above and Below the Transition Temperature. *Phys. Rev. Lett.* **17**, 1286-1289, doi:10.1103/PhysRevLett.17.1286 (1966).

9 Yuan, G. *et al.* Optical characterization of the coloration process in electrochromic amorphous and crystalline WO3 films by spectroscopic ellipsometry. *Applied Surface Science* **421**, 630-635 (2017).
